# Supplementary material for: Histone demethylase LSD1 promotes RIG-I poly-ubiquitination and anti-viral gene expression
Source: PLoS Pathog. 2021 Sep 16;17(9):e1009918. doi: 10.1371/journal.ppat.1009918 (PMC8445485; doi:10.1371/journal.ppat.1009918)
Supplement: S1 Fig — (PDF) [file ppat.1009918.s001.pdf]

S1 Fig

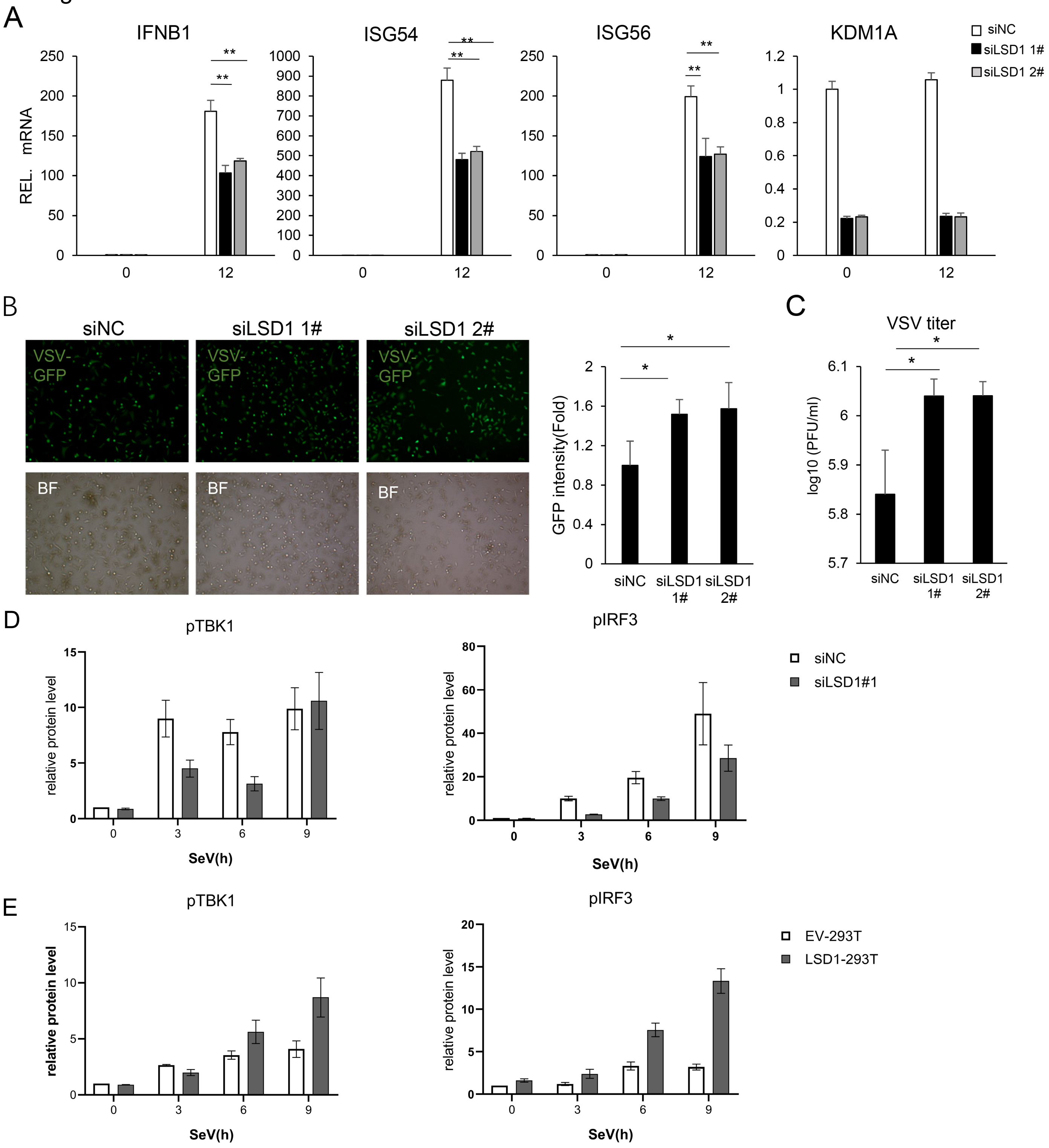

S1 Fig LSD1 regulates *IFNB1* expression in A549 cells. (A) A549 cells were transfected with negative control siRNA (siNC) or LSD1 siRNAs (1#, 2#). The cells were infected with SeV for 12h. The relative mRNA levels of IFNB1, ISG54, and ISG56 were detected by RT-qPCR. **(B)** A549 cells transfected with siNC or siLSD1 (1#, 2#) were infected with VSV-GFP (MOI=10) for 8h. The replication of VSV-GFP were observed by fluorescence microscopy (left). The intensity of GFP fluorescence were analyzed by ImageJ (right). **(C)** VSV titers in supernatants of A549 cells were tested by plaque forming unit (PFU) assay. **(D&E)** Protien amount of phosphorylated TBK1 and IRF3 in Fig. 3A (A) and 3B (B) was analyzed with ImageJ.
