# Supplementary material for: Histone demethylase LSD1 promotes RIG-I poly-ubiquitination and anti-viral gene expression
Source: PLoS Pathog. 2021 Sep 16;17(9):e1009918. doi: 10.1371/journal.ppat.1009918 (PMC8445485; doi:10.1371/journal.ppat.1009918)
Supplement: S2 Fig — (PDF) [file ppat.1009918.s002.pdf]

S2 Fig

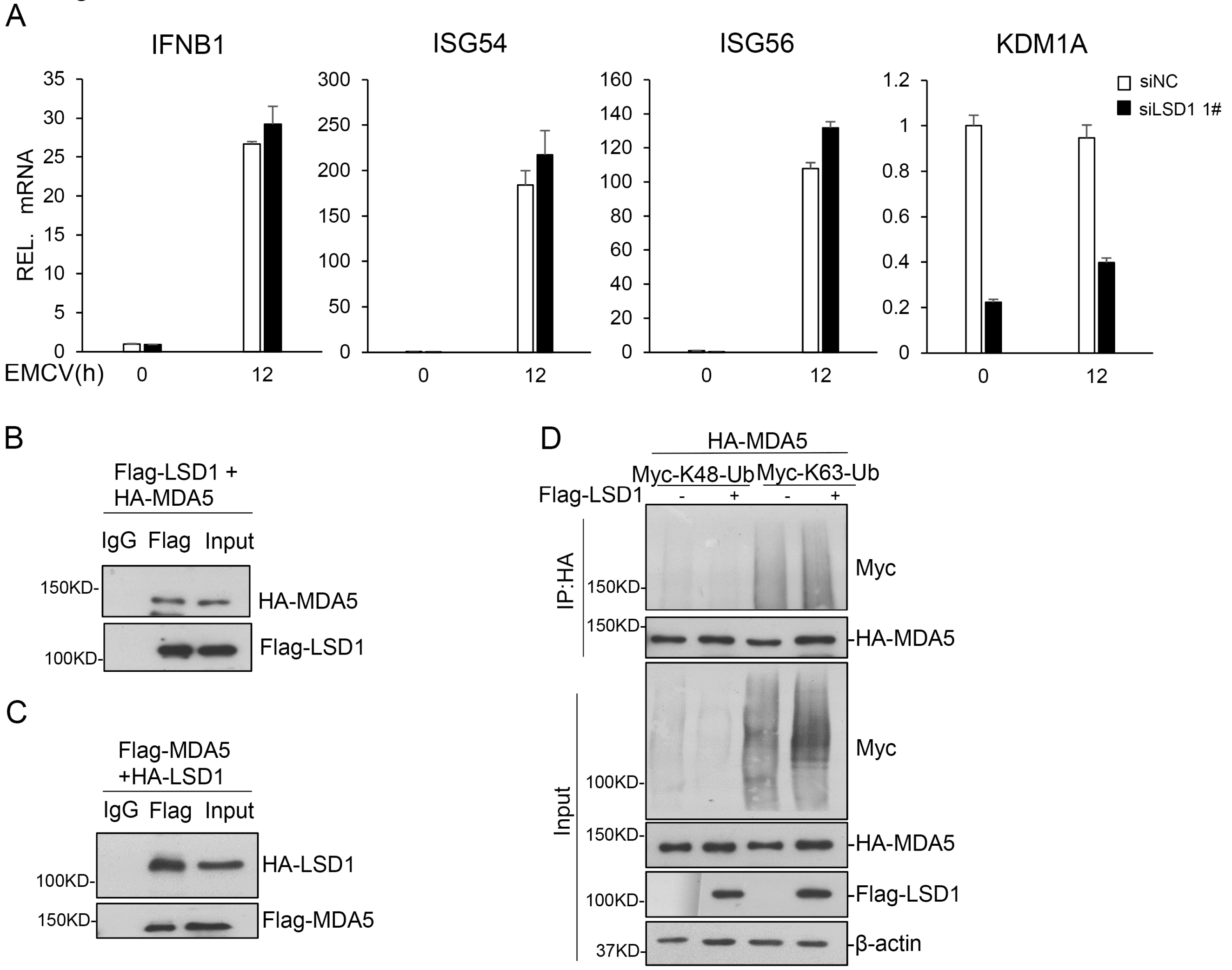

S2 Fig LSD1 is not involved in *IFNB1* expression activated by EMCV. (A) A549 cells were transfected with negative control siRNA (siNC) or LSD1 siRNAs (1#). The cells were infected with EMCV for 12h. The relative mRNA levels of *IFNB1, ISG54*, and *ISG56* were detected by RT-qPCR. **(B)** HEK293T were transfected with Flag-LSD1 and HA-MDA5 for 24h, followed by co-immunoprecipitation and immunoblotting analysis as indicated. **(C)** HEK293T were transfected with Flag-LSD1 and HA-MDA5 for 24h, followed by co-immunoprecipitation and immunoblotting analysis as indicated. **(D)** HEK293T were transfected with Myc-K63O-Ub and HA-MDA5 together with a control or Flag-LSD1 expression plasmid for 24h, followed by immunoprecipitation and immunoblotting analysis as indicated.
