# Supplementary material for: Histone demethylase LSD1 promotes RIG-I poly-ubiquitination and anti-viral gene expression
Source: PLoS Pathog. 2021 Sep 16;17(9):e1009918. doi: 10.1371/journal.ppat.1009918 (PMC8445485; doi:10.1371/journal.ppat.1009918)
Supplement: S3 Fig — (PDF) [file ppat.1009918.s003.pdf]

S3 Fig

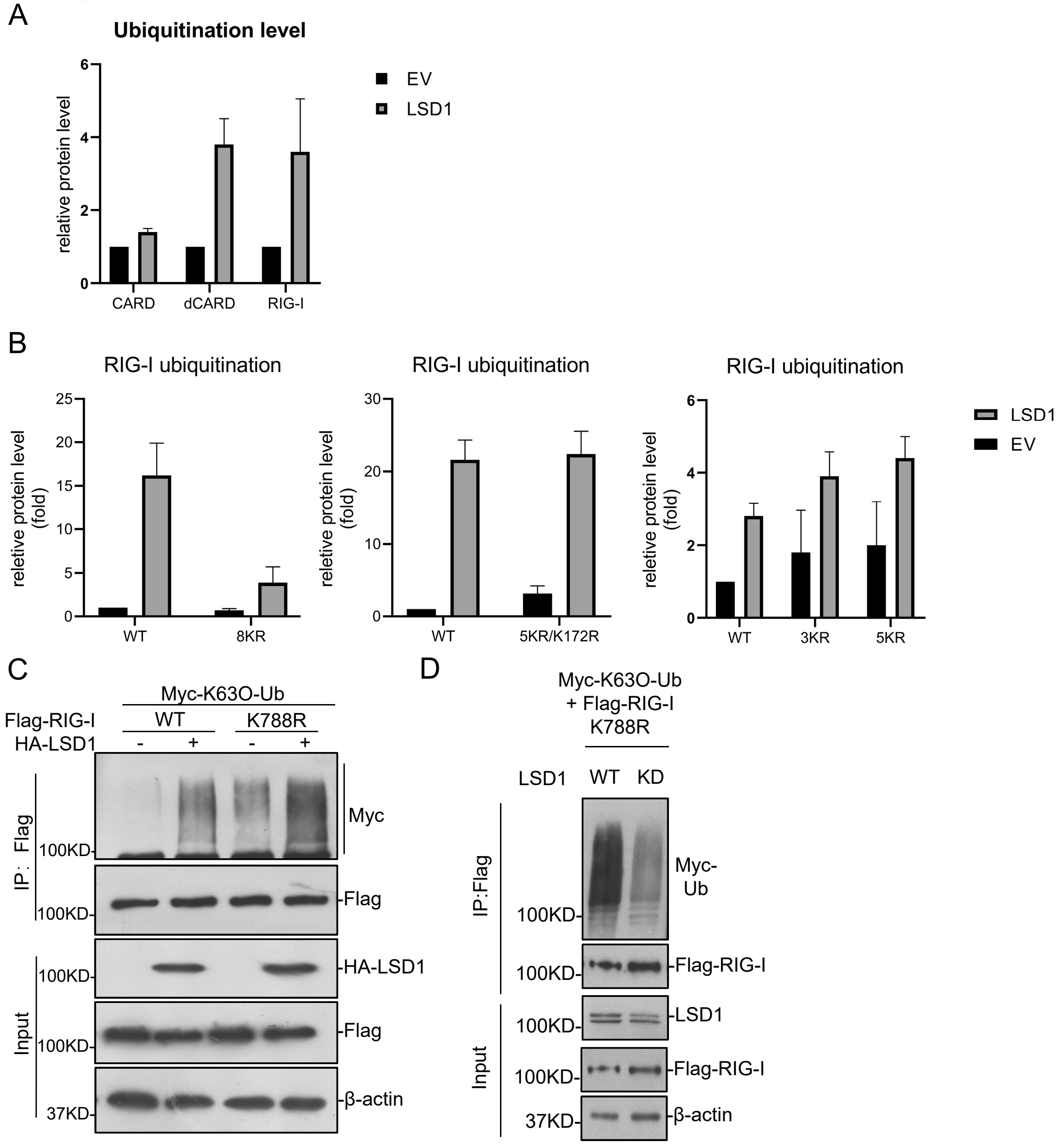

S3 Fig Functional studies of LSD1 truncations. (A) The relative RIG-I polyubiquitination level in Fig. 4E. **(B)** The relative polyubiquitination level of RIG-I mutants in Fig. 4F. **(C)** Myc-K63O-Ubiquitin and HA-LSD1 were expressed in HEK293T cell with RIG-I wild type or K788R mutant. Ubiquitination of Flag-RIG-I was analyzed. **(D)** Myc-K63O-Ub and Flag-RIG-I (K788R) were expressed in LSD1 WT or knockdown cells, and RIG-I (K788R) poly-ubiquitination was studied.
