# Supplementary material for: Histone demethylase LSD1 promotes RIG-I poly-ubiquitination and anti-viral gene expression
Source: PLoS Pathog. 2021 Sep 16;17(9):e1009918. doi: 10.1371/journal.ppat.1009918 (PMC8445485; doi:10.1371/journal.ppat.1009918)
Supplement: S4 Fig — (PDF) [file ppat.1009918.s004.pdf]

S4 Fig

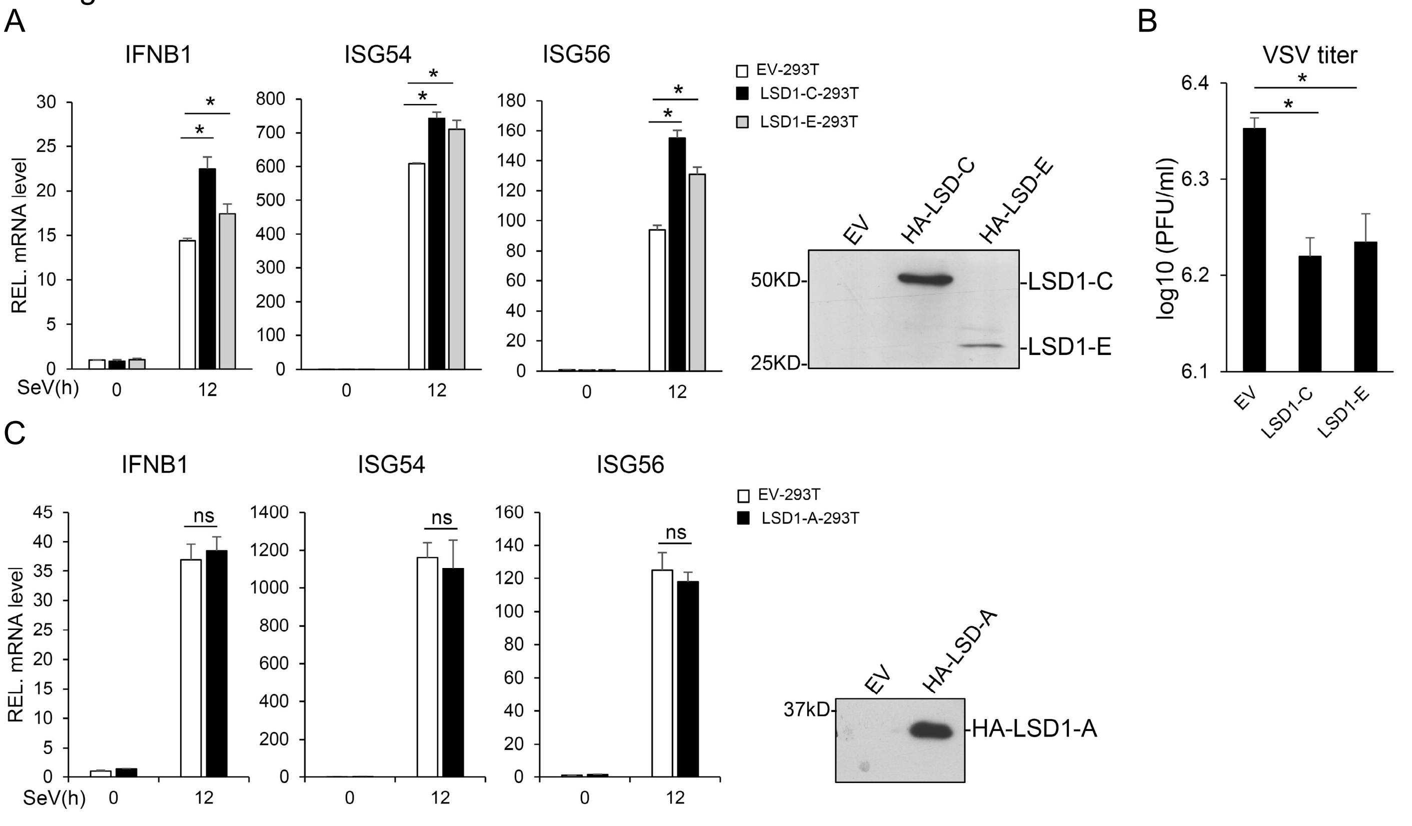

S4 Fig Functions of LSD1 truncations. (A) LSD1-C-293T, LSD1-E-293T and control cells were treated with SeV for 8 hr. The relative mRNA levels of *IFNB1*, *ISG54*, and *ISG56* were detected by RT-qPCR. Expressions of LSD1 truncations were detected by western blotting. **(B)** LSD1-C-293T, LSD1-E-293T and control cells were infected with VSV-GFP (MOI=1) for 8h. VSV titers in supernatants of 293T cells were tested by plaque forming unit (PFU) assay. **(C)** LSD1-A-293T and control cells were treated with SeV for 8 hr. The mRNA levels of *IFNB1*, *ISG54*, and *ISG56* were detected by RT-qPCR. Expressions of LSD1-A were examined by western blotting.
