# Supplementary material for: Histone demethylase LSD1 promotes RIG-I poly-ubiquitination and anti-viral gene expression
Source: PLoS Pathog. 2021 Sep 16;17(9):e1009918. doi: 10.1371/journal.ppat.1009918 (PMC8445485; doi:10.1371/journal.ppat.1009918)
Supplement: S5 Fig — (PDF) [file ppat.1009918.s005.pdf]

S5 Fig

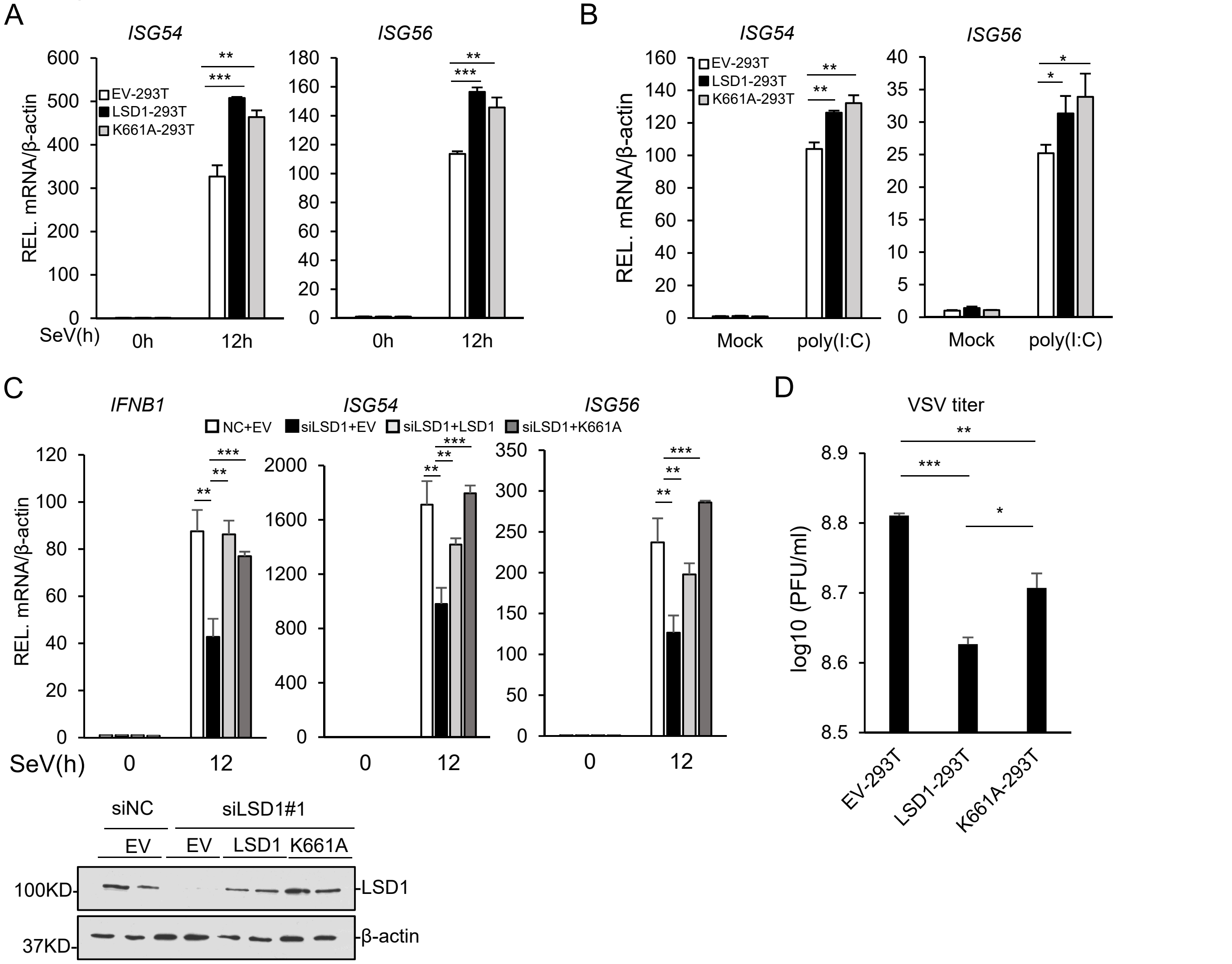

S5 Fig LSD1's function is independent of demethylase activity. (A) EV-293T, LSD1-293T and K661A-293T cells were infected with SeV for 12h. The relative mRNA levels of ISG54, and ISG56 were detected by RT-qPCR. **(B)** EV-293T, LSD1-293T and K661A-293T cells were transfected with poly(I:C) for 10h. The relative mRNA levels of ISG54, and ISG56 were detected by RT-qPCR. **(C)** HEK293T cells were lipo-transfected with siNC or LSD1 siRNA. After 12h, HEK293T cells were transfected with LSD1 or K661A plasmids for 24h and then infected by SeV for 12h. The relative mRNA levels of ISG54, and ISG56 were detected by RT-qPCR. **(D)** EV-293T, LSD1-293T and K661A-293T cells were infected with VSV-GFP (MOI=1) for 8h. VSV titers in supernatants of the HEK293T cells were tested by plaque forming unit (PFU) assay. Data are means ± SD and are representative of three independent experiments. Student's t test was used for statistical calculation. ns, no significance. *P < 0.05, **P < 0.01, and ***P < 0.001.
