# Supplementary material for: Histone demethylase LSD1 promotes RIG-I poly-ubiquitination and anti-viral gene expression
Source: PLoS Pathog. 2021 Sep 16;17(9):e1009918. doi: 10.1371/journal.ppat.1009918 (PMC8445485; doi:10.1371/journal.ppat.1009918)
Supplement: S6 Fig — (PDF) [file ppat.1009918.s006.pdf]

S6 Fig

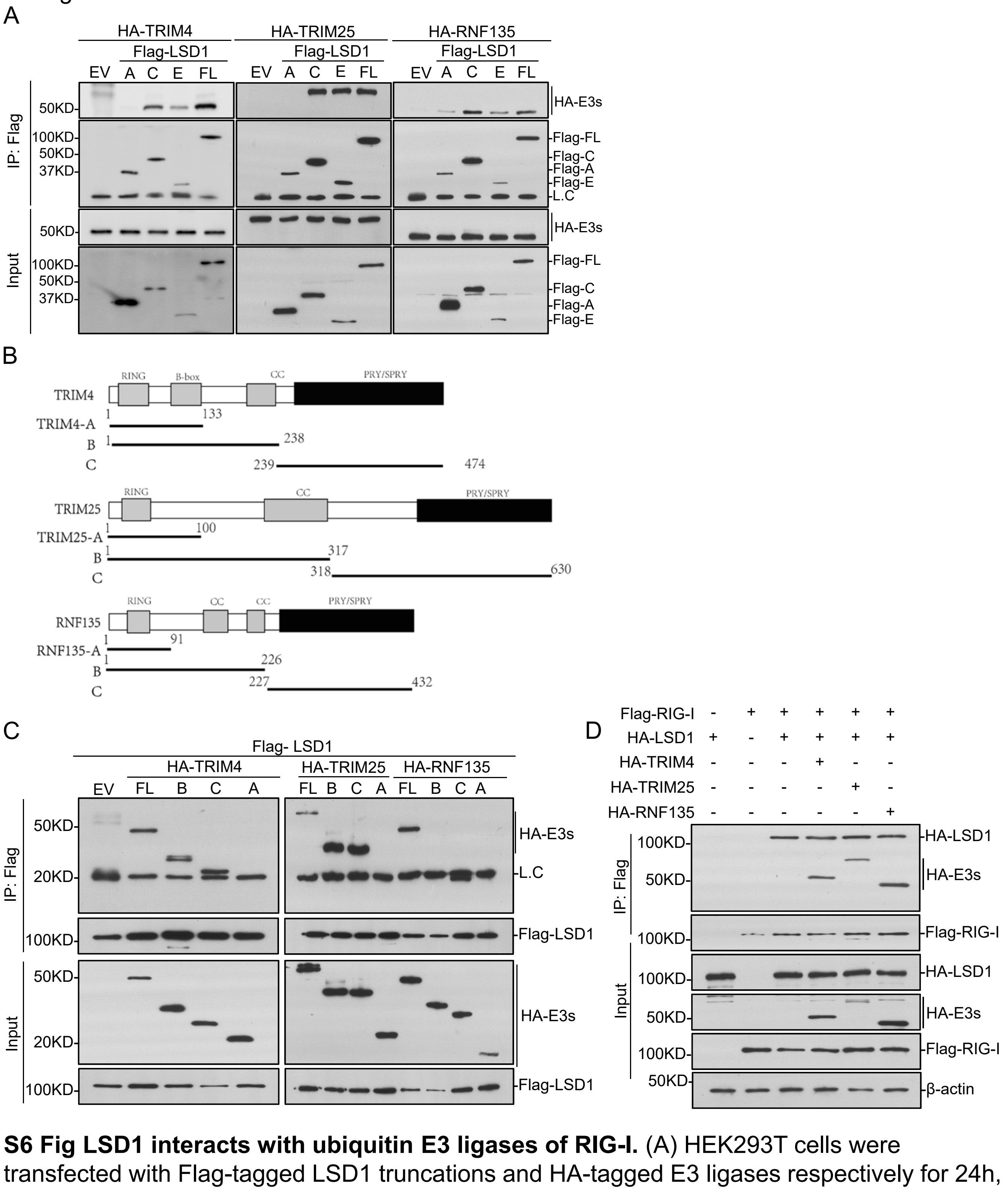

S6 Fig LSD1 interacts with ubiquitin E3 ligases of RIG-I. (A) HEK293T cells were transfected with Flag-tagged LSD1 truncations and HA-tagged E3 ligases respectively for 24h, followed by co-immunoprecipitation and immunoblotting analysis. **(B)** Schematic drawings of E3 ligases for RIG-I and their truncated mutants. **(C)** HEK293T cells were transfected with Flag-tagged LSD1 and three kinds of HA-tagged E3 ligases truncations respectively for 24h, followed by co-immunoprecipitation and immunoblotting analysis. **(D)** HEK293T cells were transfected with the indicated plasmids for 24h and interaction between RIG-I and LSD1 were examined with co-immunoprecipitation.
