# Supplementary material for: Histone demethylase LSD1 promotes RIG-I poly-ubiquitination and anti-viral gene expression
Source: PLoS Pathog. 2021 Sep 16;17(9):e1009918. doi: 10.1371/journal.ppat.1009918 (PMC8445485; doi:10.1371/journal.ppat.1009918)
Supplement: S7 Fig — (PDF) [file ppat.1009918.s007.pdf]

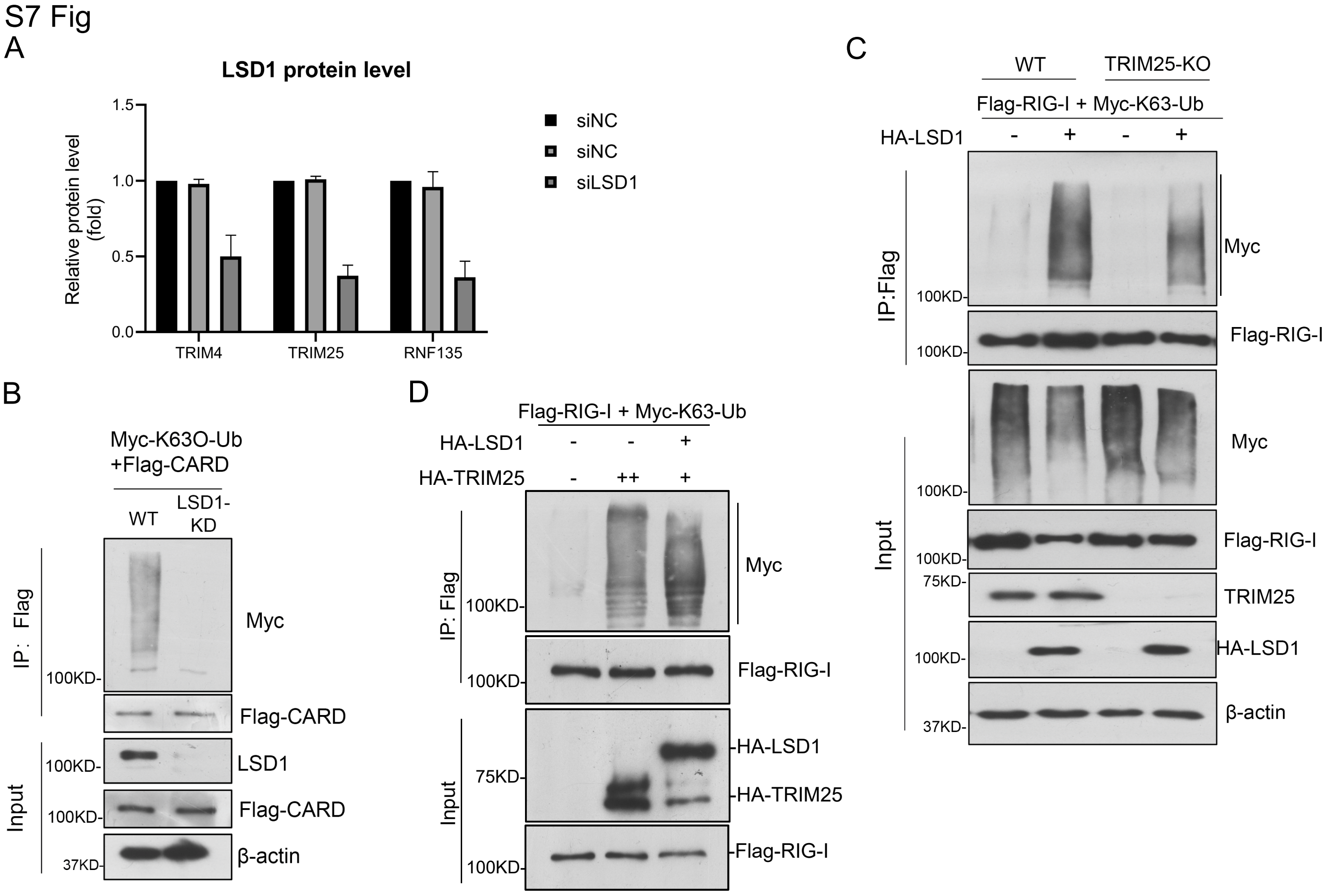


**S7 Fig LSD1 mediates TRIM25-dependent RIG-I polyubiquitination. (A)** The relative protein amount of LSD1 in cell lysates of Fig. 5F. **(B)** Myc-Ub-K63O and Flag-CARD were co-expressed in HEK293T wild type or LSD1 knockdown cells. Ubiquitination assay was then performed. **(C)** Wild-type and TRIM25-KO HEK293T cells were transfected with Myc-K63O-Ub and Flag-RIG-I together with a control or HA-LSD1 expression plasmid for 24h, followed by immunoprecipitation and immunoblotting analysis as indicated. **(D)** HEK293T were transfected with indicated plasmids for 24h, followed by immunoprecipitation and immunoblotting analysis.
