# Supplementary material for: Histone demethylase LSD1 promotes RIG-I poly-ubiquitination and anti-viral gene expression
Source: PLoS Pathog. 2021 Sep 16;17(9):e1009918. doi: 10.1371/journal.ppat.1009918 (PMC8445485; doi:10.1371/journal.ppat.1009918)
Supplement: S8 Fig — (PDF) [file ppat.1009918.s008.pdf]

S8 Fig

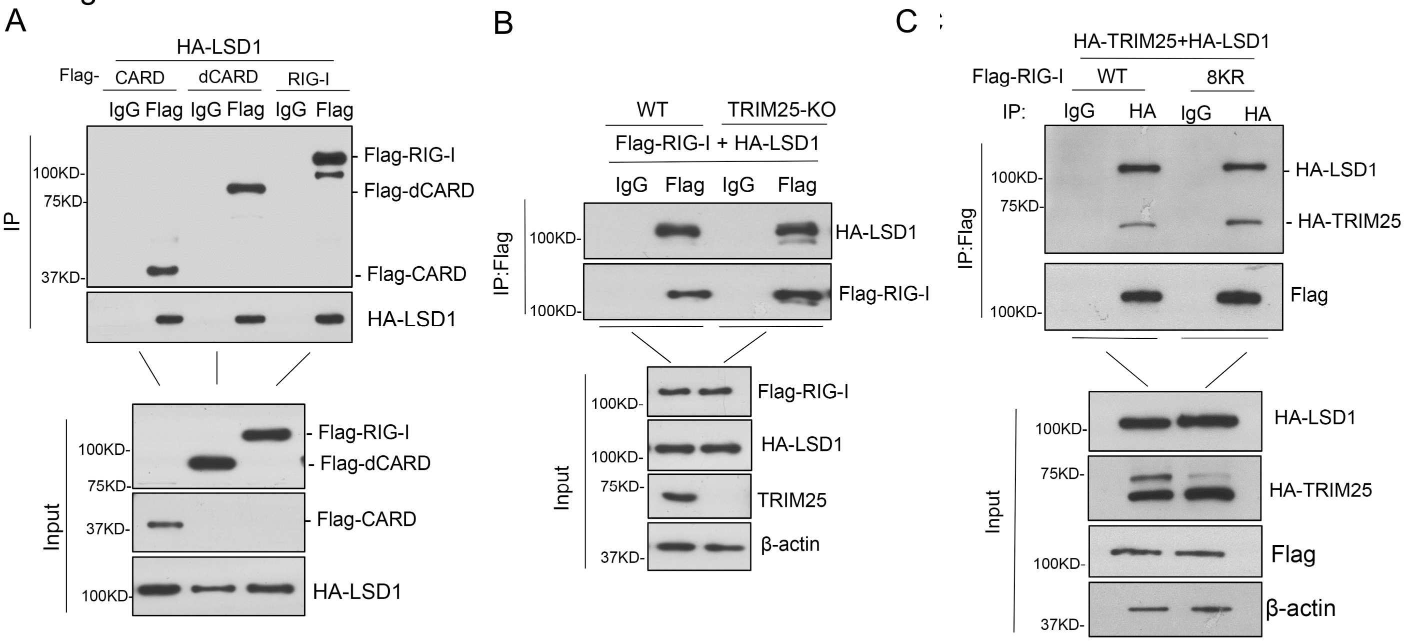

S8 Fig LSD1 interacts with RIG-I independent of TRIM25. (A) HEK293T cells were transfected with Flag-tagged RIG-I full length or truncations and HA-tagged LSD1 for 24h, followed by co-immunoprecipitation and immunoblotting analysis with indicated Abs. **(B)** Wild-type and TRIM25-KO HEK293T cells were transfected with Flag-RIG-I and HA-LSD1 for 24h, followed by co-immunoprecipitation and immunoblotting analysis with indicated Abs. **(C)** HEK293T cells were transfected with HA-tagged TRIM25 and LSD1 together with Flag-tagged RIG-I WT or 8KR mutant for 24h, followed by co-immunoprecipitation and immunoblotting as indicated.
