# Supplementary material for: Histone demethylase LSD1 promotes RIG-I poly-ubiquitination and anti-viral gene expression
Source: PLoS Pathog. 2021 Sep 16;17(9):e1009918. doi: 10.1371/journal.ppat.1009918 (PMC8445485; doi:10.1371/journal.ppat.1009918)
Supplement: S9 Fig — (PDF) [file ppat.1009918.s009.pdf]

S9 Fig

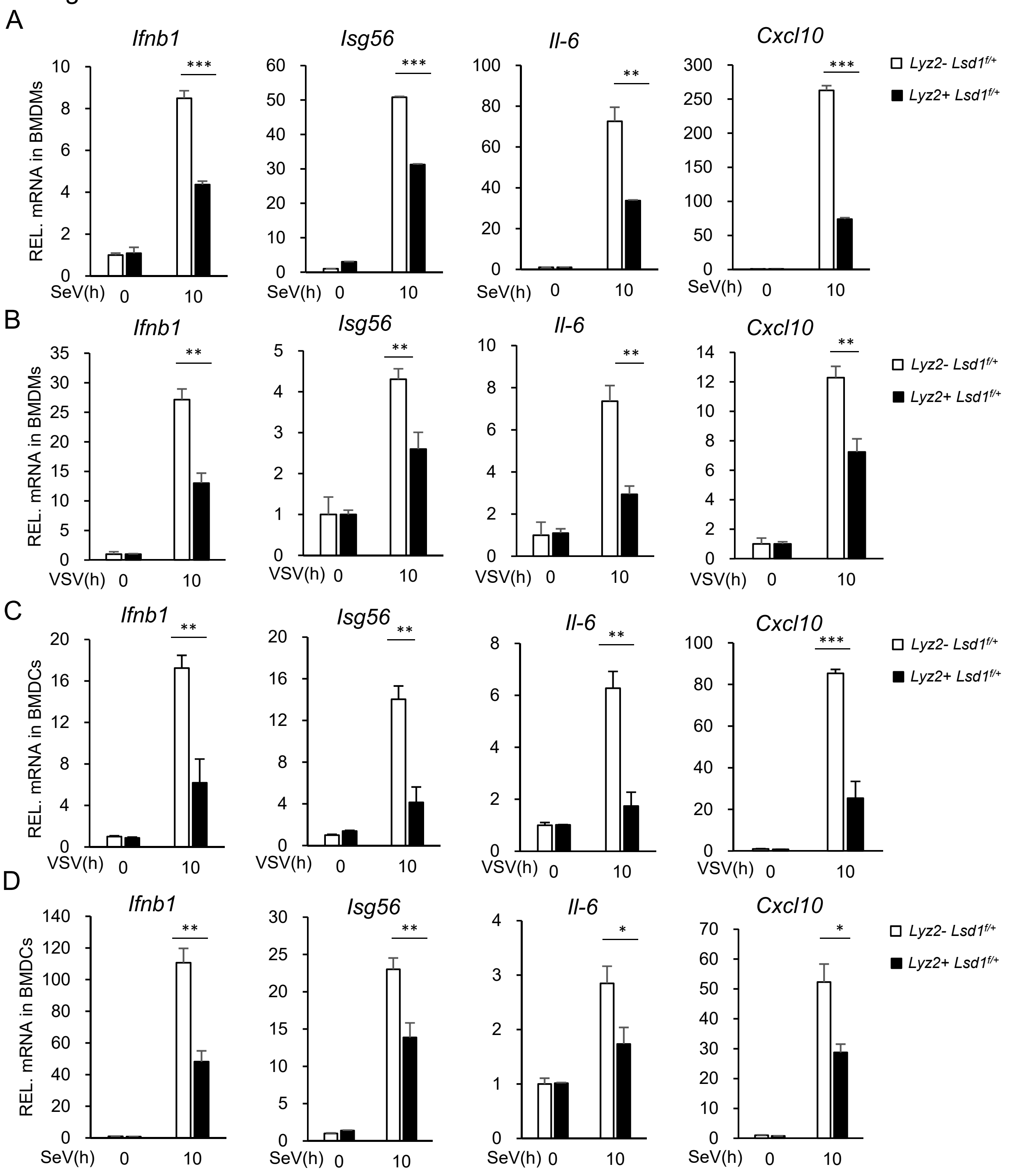

A
B
C
D
Ifnb1
Isg56
Il-6
Cxcl10
REL. mRNA in BMDMs
REL. mRNA in BMDCs
SeV(h)
VSV(h)
0
10
***
**
*
Lyz2- Lsd1f/+
Lyz2+ Lsd1f/+

S9 Fig, continued

E

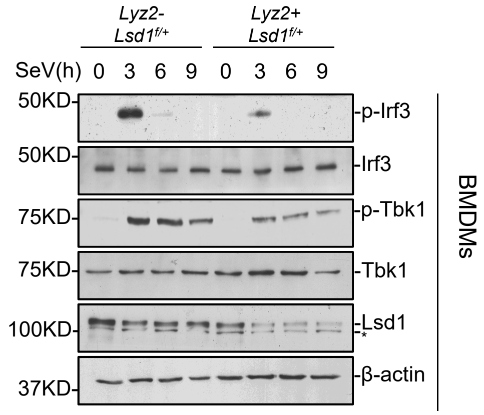

F

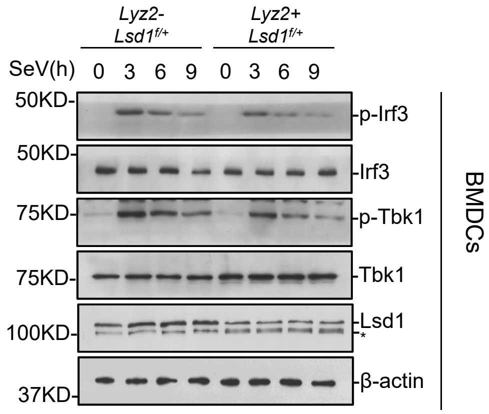

S9 Fig Effects of *Lsd1*-deficiency on RIG-I signaling in murine primary cells. (A&B) Lsd1 deficiency impairs virus-induced transcription of downstream genes in BMDMs. *Lyz2*- *Lsd1*$^{fl/+}$ and *Lyz2*+ *Lsd1*$^{fl/+}$ BMDMs were left uninfected or infected with SeV(A) or VSV(B) for 10h. The relative mRNA levels of *Ifnb1*, *Isg56*, *Il-6*, *Cxcl10* were detected by RT-qPCR. **(C&D)** Lsd1 deficiency impairs virus-induced transcription of downstream genes in BMDCs. *Lyz2*- *Lsd1*$^{fl/+}$ and *Lyz2*+ *Lsd1*$^{fl/+}$ BMDCs were left uninfected or infected with SeV(C) or VSV(D) for 10h. The relative mRNA levels of *Ifnb1*, *Isg56*, *Il-6*, *Cxcl10* were detected by RT-qPCR. **(E&F)** Lsd1 deficiency inhibits virus-induced phosphorylation of Tbk1, Irf3 in MLFs. *Lyz2*- *Lsd1*$^{fl/+}$ and *Lyz2*+ *Lsd1*$^{fl/+}$ MLFs were left un-infected or infected with SeV for the indicated times, followed by immunoblotting analysis with indicated Abs. Data are means ± SD and are representative of three independent experiments. Student's *t* test was used for statistical calculation. ns, no significance. *$P < 0.05$, **$P < 0.01$, and ***$P < 0.001$.
