# Supplementary material for: Histone demethylase LSD1 promotes RIG-I poly-ubiquitination and anti-viral gene expression
Source: PLoS Pathog. 2021 Sep 16;17(9):e1009918. doi: 10.1371/journal.ppat.1009918 (PMC8445485; doi:10.1371/journal.ppat.1009918)
Supplement: S10 Fig — (PDF) [file ppat.1009918.s010.pdf]

S10 Fig

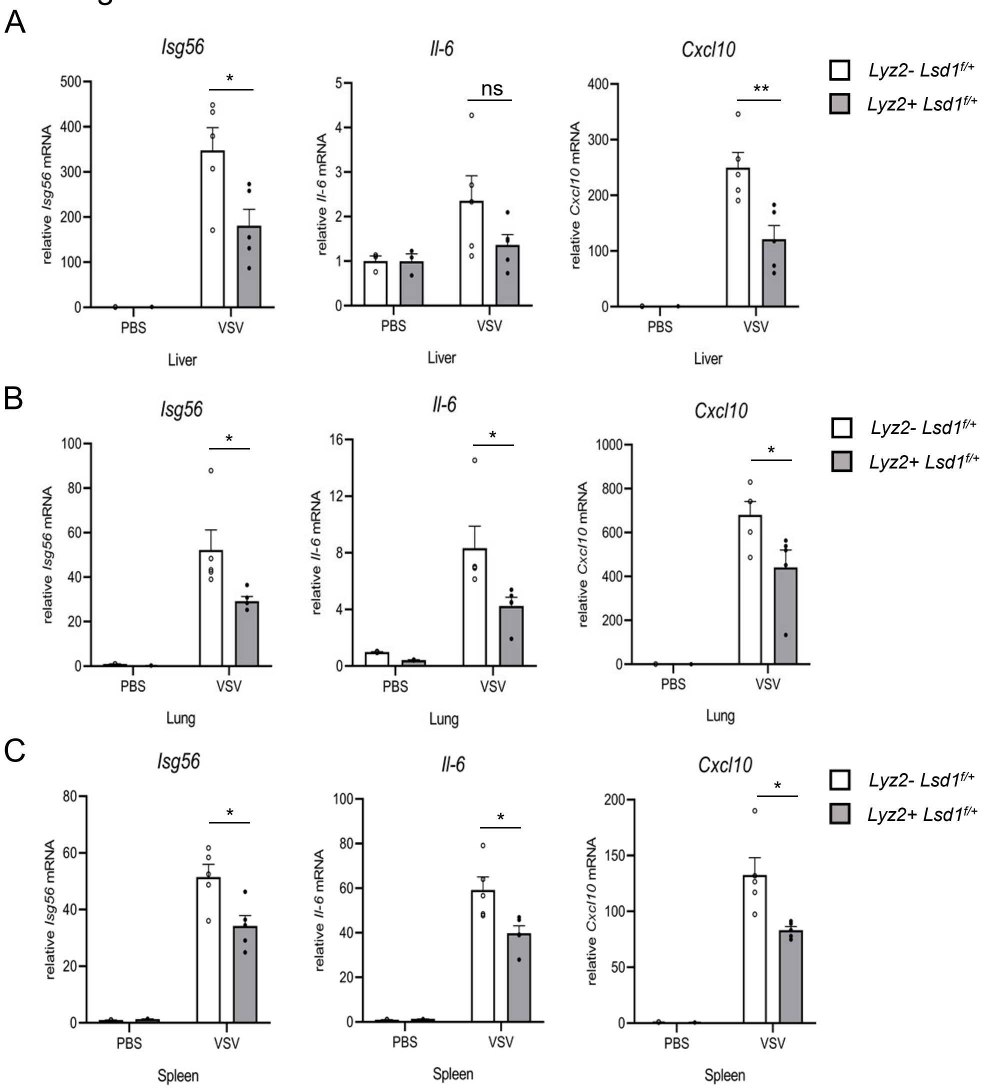

S10 Fig Lsd1 Is Required for Rig-i-Mediated Innate Immune Response in mice. (A-C) 8-week-old *Lyz2*- *Lsd1*fl/+ and *Lyz2*+ *Lsd1*fl/+ mice were tail-intravenous injected with VSV at 10^8 PFU per mouse (n = 5 for each genotype group) for 12h. Total RNA from mice liver(A), lung(B) and spleen (C) were extracted and the relative mRNA level of *Ifnb1*, *Isg56*, *Il-6*, *Cxcl10* were detected by RT-qPCR. Data are means ± SD and are representative of three independent experiments. Student's *t* test was used for statistical calculation. ns, no significance. *P < 0.05, and **P < 0.01.
