# Supplementary material for: Histone demethylase LSD1 promotes RIG-I poly-ubiquitination and anti-viral gene expression
Source: PLoS Pathog. 2021 Sep 16;17(9):e1009918. doi: 10.1371/journal.ppat.1009918 (PMC8445485; doi:10.1371/journal.ppat.1009918)
Supplement: S1 Table — (DOCX) [file ppat.1009918.s011.docx]

Table S1 . List of primers for qPCR.

| human KDM1A | Forward | GTGGACGAGTTGCCACATTTC |
| --- | --- | --- |
|  | Reverse | TGACCACAGCCATAGGATTCC |
| human IFNB1 | Forward | AAAGAAGCAGCAATTTTCAG |
|  | Reverse | CTGTCCTTGAGGCAGTATTC |
| human ISG54 | Forward | ACTGCAACCATGAGTGAGA |
|  | Reverse | ATCAAGTTCCAGGTGAAATG |
| human ISG56 | Forward | CTTTGCCTGGATGTATTACC |
|  | Reverse | AAAGCTTCTTGCAAATGTTC |
| human β-actin | Forward | CAGCACAATGAAGATCAAGA |
|  | Reverse | GATCCACATCTGCTGGAAG |
| mouse *Ifnb1* | Forward | TCCTGCTGTGCTTCTCCACCACA |
|  | Reverse | AAGTCCGCCCTGTAGGTGAGGTT |
| mouse *Isg56* | Forward | ATCGCGTAGACAAAGCTCTTC |
|  | Reverse | GTTTCGGGATGTCCTCAGTTG |
| mouse *Il-6* | Forward | TCTGCAAGAGACTTCCATCCAGTTGC |
|  | Reverse | AGCCTCCGACTTGTGAAGTGGT |
| mouse *Cxcl10* | Forward | ATCATCCCTGCGAGCCTATCCT |
|  | Reverse | GACCTTTTTTGGCTAAACGCTTTC |
| mouse *Gapdh* | Forward | ACGGCCGCATCTTCTTGTGCA |
|  | Reverse | ACGGCCAAATCCGTTCACACC |
| VSV RNA | Forward | ACGGCGTACTTCCAGATGG |
|  | Reverse | CTCGGTTCAAGATCCAGGT |
