# Supplementary material for: Histone demethylase LSD1 promotes RIG-I poly-ubiquitination and anti-viral gene expression
Source: PLoS Pathog. 2021 Sep 16;17(9):e1009918. doi: 10.1371/journal.ppat.1009918 (PMC8445485; doi:10.1371/journal.ppat.1009918)
Supplement: Attachment — Submitted filename: Response letter.docx [file ppat.1009918.s012.docx]

To whom it may concern,

Thank you for giving us a chance to submit our revised manuscript, titled “*Histone demethylase LSD1 promotes RIG-I poly-ubiquitination and anti-viral gene expression*” (PPATHOGENS-D-21-00884 - [EMID:993ca9eeae8fa665]). We have performed additional experiments to address the comments raised by the reviewers. Below is our point-by-point response to each comment, in which the text in blue is the original comments from the reviewers and our response in black.

Reviewer's Responses to Questions

Part I - Summary

Please use this section to discuss strengths/weaknesses of study, novelty/significance, general execution and scholarship.

Reviewer #1: The manuscript by Hu et al. reports the identification of histone demethylase LSD1 as a positive regulator of the RIG-I signaling pathway. Silencing and ectopic expression of LSD1 confirmed its role in regulating antiviral ISG/cytokine responses and VSV replication. Biochemical evidence showed that LSD1 interacted with RIG-I and promoted its K63-linked polyubiquitination, and this effect was independent of its demethylase activity and was likely due to promoting interaction between RIG-I and E3 ligases, particularly TRIM25. Lastly, the role of LSD1 in the regulation of cytokine responses and viral replication in vivo was corroborated by using VSV challenge of myeloid cell lineage conditional KO mice.

The manuscript extends the antiviral function of LSD1 to RLR-mediated innate immune responses and presents a good amount of data exploring the potential mechanism by which LSD1 regulates RIG-I. The study is expected to be of high interest to the field. However, some of the mechanistic insights are obscured by an abundance of negative data that were likely due to overexpression artifacts and are difficult to interpret. Therefore, a defined role for LSD1 in regulating RIG-I-mediated antiviral innate immunity should be further strengthened by additional experiments.

Response: Thanks for the positive comments of our work. We have performed additional experiments during revision to further strengthen our conclusion.

Reviewer #2: RIG-I is one of the central innate immune sensors of invading RNA (and some DNA) viruses. Tri (or di) phosphorylated RNA produced upon viral infection is recognized by RIG-I and a signaling cascade ultimately leading to the induction of type-I IFNs (via IRF3/NF-kB) and other pro-inflammatory cytokines (NF-kB) is initiated. For full activation of RIG-I it has to be post-translationally modified by K63-linked ubiquitination, which is mediated by e.g. TRIM25, RNF135, TRIM4 and/or MEX3C.

In this manuscript Hu and co-authors propose that the methyltransferase LSD1 positively regulates RIG-I signaling by enhancing the interaction of RIG-I with the E3 ligase TRIM25, which promotes K63-linked polyubiquitination and the interaction with MAVS/VISA. Accordingly, LSD1 overexpression increases SeV/poly(I:C)-induced expression of antiviral genes and inhibits replication of VSV. Knockdown experiments show the opposite effect, which was also confirmed in LSD1-deficient mice. Further, the function of LSD1 is not dependent on its methylase activity.

In summary, in an updated version this manuscript would provide novel and interesting insight into the regulation of the innate immunity sensor RIG-I. The study is well planned, defines a phenotype and novel (albeit moderate) regulator of RLR signaling -that is relevant in vivo- but the mechanistic analyses fall short and do not (yet) support the main conclusions.

Response: Thanks for the positive comments of our work. We have performed additional experiments during revision to further deepen the mechanistic insight.

Reviewer #3: The authors of this manuscript found that the histone demethylase LSD1 could interact with RIG-I, and that knockdown of LSD1 expression reduced poly(I:C) or SeV-induced IFN-beta and ISG production. In addition, overexpression of LSD1 enhanced the K63-ubiquitination of RIG-I. The authors also showed that LSD1 could be involved in the interaction between RIG-I and TRIM25. Finally, the research applied conditional LSD1 knockout mice to demonstrate the role of LSD1 in IFN-beta and antiviral response. The findings of this research are quite interesting in the research field of innate immune response. Although the authors provided many biochemical evidences to correlate LSD1 and RIG-I activation in the manuscript, several points should be further clarified before drawing the conclusion.

Response: Thanks for the positive comments of our work. We have performed additional experiments during revision to clarify the issues and further strengthen our conclusion.

Part II – Major Issues: Key Experiments Required for Acceptance

Please use this section to detail the key new experiments or modifications of existing experiments that should be absolutely required to validate study conclusions.

Generally, there should be no more than 3 such required experiments or major modifications for a "Major Revision" recommendation. If more than 3 experiments are necessary to validate the study conclusions, then you are encouraged to recommend "Reject".

Reviewer #1: 1. The silencing effect of LSD siRNA was marginal in Fig 2B (and, in fact, in many other panels), though there seemed to be a statistically significant phenotype on VSV replication (Fig 2C). Is this related to the cell type (293T) used in which endogenous LSD1 was hard to deplete? Have the authors tried other cell types such as lung epithelial cells or fibroblasts that are also more physiologically relevant to VSV infection? It appears that the entire study, except for the ex vivo data in Fig 6, was performed in HEK 293T cells. The data on the phenotype on cytokine induction and viral replication should be confirmed using at least one more relevant cell type, such as A549 cells, or primary cells preferably.

Response: In our study, we not only used siRNA, but also overexpression and knockout mice, to study the role of LSD1 in RIG-I signaling. All the data pointed to the same conclusion. During revision, we have performed the suggested experiments in A549 cells. A549 cells were transfected with negative control siRNA (siNC) or two different LSD1 siRNAs (1#, 2#), and then infected with virus. The results indicated that the expression of anti-viral genes decreased in LSD1-deficient cells, while VSV amount increased (Response Fig. 1), which was consistent with our previous conclusion.

Response Fig. 1 (A) A549 cells were transfected with negative control siRNA (siNC) or LSD1 siRNAs (1#, 2#). The cells were infected with SeV for 12h. The relative mRNA levels of IFNB1, ISG54, and ISG56 were detected by RT-qPCR. (B) A549 cells transfected with siNC or siLSD1 (1#, 2#) were infected with VSV-GFP (MOI=10) for 8h. The replication of VSV-GFP were observed by fluorescence microscopy (left). The intensity of GFP fluorescence were analyzed by ImageJ (right). (C) VSV titers in supernatants of A549 cells were tested by plaque forming unit (PFU) assay.

2. In Fig 4, the potentiating effect of LSD1 on RIG-I polyubiquitination was only examined in the context of LSD1 overexpression, which should be validated by LSD1 silencing. Specifically, since LSD1 interacted strongly with the 2CARD (Fig 3F), which is known to undergo robust K63-linked polyubiquitination without the requirement of Ub overexpression, the silencing experiment should be preferentially conducted using the 2CARD construct.

Response: In Fig. 4, because the poly-ubiquitination of 2CARD construct had very strong basal level, its elevation by LSD1 was not very clear when under long exposure. We showed the long- and short-time exposure together here (Response Fig. 2A). We have also examined poly-ubiquitination of 2CARD in LSD1 KD cells, which showed that 2CARD showed strong polyubiquitination in WT cells but not in LSD1-KD cells (Response Fig. 2B). We have repeated the experiments many times and are quite sure LSD1 exogenous expression can also elevate 2CARD polyubiquitination (Response Fig. 2C).

Response Fig. 2 (A) Long- and short-time exposure to show the poly-ubiquitination of RIG-I truncations with LSD1 exogenous expression in Fig. 4E. (B) Myc-Ub-K63O and Flag-CARD were co-expressed in HEK293T wild type or LSD1 knockdown cells. Ubiquitination assay was then performed. (C) Myc-Ub-K63O and Flag-CARD were co-expressed in HEK293T cells with or without HA-LSD1. Ubiquitination on Flag-CARD was examined.

For mapping ubiquitination sites in the context of LSD1 overexpression (Fig 4F), the experimental setting should include key E3 ligase co-expression (or stimulation with a RIG-I ligand), as there was essentially not even a difference between the WT and nKR mutants in ubiquitination levels in the absence of LSD1.

Response: In Fig. 4F, we are showing the effect of exogenous expressed LSD1 on RIG-I mutants. Since exogenous expressed LSD1 dramatically enhanced LSD1 ubiquitination without virus treatment, it was not necessary to activate RIG-I polyubiquitination using other factors. During revision, we have established a cell line with TRIM25 knockout, and our result showed that in the absence of TRIM25, RIG-I poly-ubiquitination promoted by LSD1 was greatly reduced, which further support that RIG-I polyubiquitination promoted by LSD1 is dependent on TRIM25 (Response Fig. 3).

Response Fig. 3 Wild-type and TRIM25-KO HEK293T cells were transfected with Myc-K63O-Ub and Flag-RIG-I together with a control or HA-LSD1 expression plasmid for 24h, followed by immunoprecipitation and immunoblotting analysis as indicated.

In addition to the nKR controls, the focus should be placed on K172R and K788R as both sites are the major ubiquitination sites by TRIM25 and Riplet, respectively (Gack et al. 2007; Oshiumi et al. 2013).

Response: For K172R mutant, we have provided results in our manuscript (Fig. 4F&5G). In the manuscript, since we have shown that endogenous LSD1 mainly regulates the function of TRIM25, we did not put much effort on other sites. During revision, we examined K788R mutant in LSD1-mediated RIG-I ubiquitination. The results showed that LSD1 exogenous expression still promoted poly-ubiquitination of K788R mutant, while its poly-ubiquitination was down-regulated in LSD1 KD cells (Response Fig. 4A&B). These indicated that polyubiquitination promoted by LSD1 is not dependent on K788.

Response Fig. 4 (A) Myc-K63O-Ubiquitin and HA-LSD1 were expressed in HEK293T cell with RIG-I wild type or K788R mutant. Ubiquitination of Flag-RIG-I was analyzed. (B) Myc-K63O-Ub and Flag-RIG-I (K788R) were expressed in LSD1 WT or knockdown cells, and RIG-I (K788R) poly-ubiquitination was studied.

A limiting concentration of LSD1 might also have to be experimentally determined so that it does not overwhelm the system thereby masking any effect on the KR mutants (see also point #5).

Response: In Fig. 4C, we have performed a dose assay for LSD1. Three different amounts of LSD1 plasmids were used in the assay, 0.3 μg, 0.8 μg and 1.5 μg per well of a 6-well plate. Then we were using 0.8 μg for most of the experiments.

3. The data in Fig 5G are somewhat misleading as WT RIG-I was the least ubiquitinated compared to other KR mutants; samples should be loaded on the same gel for an unbiased comparison. Nevertheless, Fig 5G does clearly point to TRIM25 as the LSD1 target and the K172 as the major site of ubiquitination promoted by LSD1. However, how do these data reconcile with that in Fig 4F then? As the authors were proposing an interesting model where LSD1 potentially bridges the interaction between TRIM25 and RIG-I, additional data showing that LSD1 can sensitize a limiting amount of TRIM25 to catalyze RIG-I ubiquitination to a similar level as does a high concentration of TRIM25 only would further strengthen their proposed model.

Response: The purpose of Fig. 5G was mainly to compare whether LSD1 knockdown impaired the polyubiquitination of different RIG-I constructs. When performing the experiment, we found it was difficult to handle such as a large set of plasmids and control their expression level to a similar level. To better clarify the effect of LSD1, we chose to examine each mutant in individual experiment. We hope the reviewer could understand the difficulty of the experiment.

During revision, we performed the suggested experiment. LSD1 was co-expressed with a low amount of TRIM25, which was compared with another sample with high expression of TRIM25. The results showed that TRIM25 increased RIG-I poly-ubiquitination, as previously reported; while LSD1 and low amount TRIM25 potentiated RIG-I poly-ubiquitination to the similar level as high amount TRIM25 (Response Fig. 5).

Response Fig. 5 HEK293T were transfected with indicated plasmids for 24h, followed by immunoprecipitation and immunoblotting analysis.

Reviewer #2: In general, the effects of LSD1 are very moderate. The authors could consider using LSD1 KO cells, if feasible for their experiments.

Response：In the study, we found that LSD1 knockout cell lines were not reliable and different KO clones gave us different results, which were not always consistent with LSD1 knockdown or over expression. It is maybe because that LSD1 controls transcription of many genes and its complete knockout had a big impact to cells; during selection, some clones somehow changed to adapt to the LSD1 deficient status. As comparison, the results of transient knockdown with siRNA, sgRNA knockdown with a short-time selection, over expression and heterozygous KO mice were all consistent with our conclusion. Moreover, one study showed that long-time LSD1 inhibition could increase the expression of transposons and activate inflammation genes (1), which might interfere with our observation.

Fig. 3: LSD1 was described as mainly, if not exclusively nuclear. How is an association with the cytoplasmic RIG-I/TRIM25 complex rationalized? The authors need to show that in a cell LSD1/RIG-I/TRIM25 can be found in a similar localization. This issue has to be addressed experimentally.

Response: We expressed HA-RIG-I and Flag-LSD1 in cells and performed immune-staining. The result showed that in some cells, LSD1 mainly was localized in nuclei; and in others, LSD1 was both in nuclei and cytoplasm (Response Fig. 6). It suggests that LSD1 probably can translocate into cytoplasm under certain conditions.

Response Fig. 6 (A) HA-RIG-I and Flag LSD1 were expressed in cells, and immunofluorescent staining was performed with antibodies recognizing Flag and HA. (B) HA-TRIM25 and Flag LSD1 were expressed in cells and experiment was performed as (A).

Fig. 4A: Ubiquitination of RIG-I is dramatically increased by LSD1 expression; however, it seems to not activate RIG-I as in cells overexpression of LSD1 does not stimulate ISG expression. Compare also Fig. 5C. Can the authors explain why this is the case, and whether the ubiquitination of RIG-I they observe then even contributes to RIG-I activation?

Response: We also noticed the result. It indicated that RIG-I poly-ubiquitination alone is not sufficient to activate the pathway, and some other events are required, which is interesting to be studied in the future.

Fig 4E/F and text lines 255-256: Text and Fig 4E and 4F are not consistent. 4E: It does not look like LSD1 promotes Flag-CARD ubiquitination, the levels seem to be equal with and without LSD1.

Response: We have checked the manuscript and corrected the mistake. The ubiquitination of Flag-CARD here was over exposed. We have shown a short-exposed picture here (Fig. 4E, Response Fig 2), and added it into the manuscript. Since Flag-CARD has very strong basal level of ubiquitination than others, sometimes it was difficult to show them together in one blot.

In contrast 4F indicates that LSD1 ubiquitinates CARD (3KR) and dCARD (5KR) to comparable levels. How do the authors explain that the classically by TRIM25 ubiquitinated lysines can be removed and still enhanced ubiquitination is observed for LSD1 overexpression if its mechanism is TRIM25 dependent? Fig. 4E: How can ubiquitination of RIG-IdCARD be promoted if RIG-IdCARD does not interact with LSD1? The passage in the manuscript (lines 255/256) states that 4E is consistent with the IP from Fig 3F, which is not the case. The current inconsistencies suggest to me that the proposed mechanism is not completely correct. The authors need to provide more evidence to support their conclusions and explain why some of the current data is not supporting this hypothesis. For example: In TRIM25KO cells, does LSD1 still promote ubiquitination of RIG-I? Is the RIG-I/TRIM25/LSD1 complex formed in the absence of RIG-I ubiquitination (e.g. using the 8R mutant).

Response: As we stated in the manuscript, when LSD1 was over expressed, it facilitated the interaction between RIG-I and all tested E3 ligases; while the endogenous LSD1 is mainly required for RIG-I ubiquitination promoted by TRIM25. In Fig. 4F, although K172 was mutated, overexpressed LSD1 also promoted ubiquitination by E3s other than TRIM25.

About the IP result in Fig. 3F, although LSD1 pulled down much less Flag-dCARD than Flag-CARD, but it was still comparable with the full-length RIG-I. During revision, we repeated the experiments and got the same conclusion (Response Fig. 7A). Since over-expressed LSD1 could interact with both CARD, dCARD and RIG-I, we thought it was consistent with Fig. 4E, which showed that overexpressed LSD1 promoted ubiquitination of Flag-CARD, dCARD, and RIG-I. These results still support our hypothesis.

Nevertheless, we still performed the suggested experiments. The results showed that in TRIM25 KO cells, LSD1 still interacted with RIG-I (Response Fig. 7B), while RIG-I poly ubiquitination promoted by LSD1 was greatly repressed (Response Fig. 7C). The 8KR RIG-I mutant could still interact with LSD1 and TRIM25.

Response Fig. 7 (A) HEK293T cells were transfected with Flag-tagged RIG-I full length or truncations and HA-tagged LSD1 for 24h, followed by co-immunoprecipitation and immunoblotting analysis with indicated Abs. (B) Wild-type and TRIM25-KO HEK293T cells were transfected with Flag-RIG-I and HA-LSD1 for 24h, followed by co-immunoprecipitation and immunoblotting analysis with indicated Abs. (C) HEK293T cells were transfected with HA-tagged TRIM25 and LSD1 together with Flag-tagged RIG-I WT or 8KR mutant for 24h, followed by co-immunoprecipitation and immunoblotting as indicated.

Fig. 4: For all ubiquitination assays please show the input myc (ubiquitin) blots. This is mandatory to exclude differences in the input levels that could affect the IP.

Response: To make sure to add equal amount of plasmids during transfection, we always pre-mixed the plasmids together and then aliquoted them into different tubes. We tend to use other proteins as controls because proteins bands are much better than ubiquitination smears for comparison. During revision, we measured the myc-Ub level for some extracts (Fig. 4B, Response Fig. 3, 8 & 11). We could not do it for all experiments, otherwise we were unable to finish the revision within the requested time.

Reviewer #3: 1. The authors showed that the expression of IFN-beta and ISGs was changed by knockdown or overexpression of LSD1 in the Figure 1. However, it cannot conclude that “LSD1 plays an important role in promoting RIG-I signaling” (line 194) from the results. From this part of the results, it can only conclude that LSD1 is involved in regulation of IFN-beta and ISG expression.

Response: We have changed the text according to the suggestion.

2. From the results in Figure 3, the authors could only conclude that LSD1 could affect TBK1 and IRF3 phosphorylation, not really “functions upstream of TBK1” (lines 214-215), unless the authors determine the LSD1’s role in IFN-beta expression under TBK1 overexpression.

Response: We have changed the text according to the suggestion.

3. The results showed that the endogenous LSD1 interacts with RIG-I even at “0 h” after SeV infection (Fig. 3D, lane 2). Does this mean that endogenous LSD1 could interact with RIG-I and facilitate RIG-I K63 ubiquitination/activation without any stimulation? However, overexpression of LSD1 itself seems not to activate IFN-beta (Fig. 1F). The authors have to explain the controversial results.

Response: Yes, our data indicated that LSD1 interacts with RIG-I without virus treatment, and it promoted RIG-I poly-ubiquitination but did not activate IFNB expression without virus. These indicated that RIG-I polyubiquitination activated by LSD1 alone is not sufficient to activate the pathway, and something else is required. We have added these into the Discussion section.

4. The results for determining the region of LSD1 for RIG-I interaction showed that multiple regions in LSD1 can interact with the RIG-I (Fig. 3H). Does overexpression of each LSD1 construct (B-E) enhance IFN-beta/ISG expression under poly(I:C) or SeV stimulation and suppress viral replication?

Response: We have checked the function of LSD1 truncation A/C/E during revision. As seen in Response Fig. 8, LSD1-C and LSD1-E expression enhanced the induction of *IFNB1, ISG54* and *ISG56*, and repressed VSV replication. As comparison, LSD1-A did not enhanced expression of the above genes. As shown in Fig. 4G, when exogenous expressed in cells, LSD1-C and -E promoted RIG-I poly-ubiquitination as LSD1 full length, but not LSD1-A. These indicated that the function of LSD1 truncations in activating *IFNB1* expression is probably associated with their ability in regulating RIG-I ubiquitination.

Response Fig. 8 (A) LSD1-C-293T, LSD1-E-293T and control cells were treated with SeV for 8 hr. The relative mRNA levels of *IFNB1*, *ISG54*, and *ISG56* were detected by RT-qPCR. Expressions of LSD1 truncations were detected by western blotting. (B) LSD1-C-293T, LSD1-E-293T and control cells were infected with VSV-GFP (MOI=1) for 8h. VSV titers in supernatants of 293T cells were tested by plaque forming unit (PFU) assay. (C) LSD1-A-293T and control cells were treated with SeV for 8 hr. The mRNA levels of *IFNB1*, *ISG54*, and *ISG56* were detected by RT-qPCR. Expressions of LSD1-A were examined by western blotting.

5. The construct B of the truncated LSD1 interacts with RIG-I well (Fig. 3H). However, the truncated LSD1 does not enhance much K63 ubiquitination (Fig. 4G). The authors should explain this phenomenon.

Response: The results suggested that the N-terminal part of LSD1 AOL domain contains the sequence for RIG-I interaction, but not that for RIG-I polyubiquitination; while the Tower domain and C terminal part of AOL domain are critical for RIG-I ubiquitination.

6. The conclusion described in lines 229-230 should be modified, unless the authors can map the site in LSD1 for RIG-I interaction and show that the RIG-I binding site mutant of LSD1 loses the ability for stimulating RIG-I polyubiquitination.

Response: We have changed the text according to the suggestion.

7. In the Fig. 4E, the overexpression of LSD1 did not enhance polyubiquitiation of RIG-I-CARDs (Fig 4E, lane 1 vs 2). It has been known that the ubiquitination of K172 in RIG-I CARDs is mainly mediated by TRIM25. The result showed that LSD1 could interact with TRIM25 (Fig. 5D and E). Why LSD1 overexpression did not enhance polyubiquitiation of RIG-I-CARDs?

Response: Flag-CARD has strong signal of basal ubiquitination level, which makes it difficult to tell the difference. We have provided the short-exposure picture of the same experiment, which showed that LSD1 can promote poly-ubiquitination of Flag-CARD (Response Fig. 2). Moreover, we repeated the experiment during revision, which showed the same conclusion (Response Fig. 2B&C).

8. In Supp Fig. 1C, the knockdown efficiency of LSD1 looks perfect (no LSD1 was detected in lanes 3 and 4), not like in the Fig. 1C, 2B and 3A. However, the reduction of SeV-activated IFN-beta/ISG expression looks similar. The authors may have to perform a dose-dependent experiment (knockdown or overexpression of LSD1) to conclude that LSD1 is involved in regulation of IFN activation.

Response: In Fig. 4C, we have shown that LSD1 promotes K63-linked RIG-I polyubiquitination in a dose dependent manner. Actually, it is quite normal in different experiments, the density of WB bands was different because of different exposure time, and it is not appropriate to compare the knockdown efficiency of different experiments based on the band density. Nevertheless, we performed one dose experiment using knockdown, control and over-expression of LSD1 (Response Fig. 9). The result clearly showed that LSD1 regulates the expression of IFNB1, ISG54 and ISG56 in a dose-dependent manner.

Response Fig. 9 wild type, LSD1 knockdown and over-expressed 293T cells were treated with SeV for 10 hr, and the indicated genes were assayed by quantitative RT-PCR. * stands for p value < 0.05; KD for knockdown, EV for empty vector and OE for over-expression.

9. In the Fig. 5F, the knockdown efficiency was around 50%. However, the TRIM25-RIG-I interaction was almost abolished (lane 5 vs 6). Could the authors explain it?

Response: We have replaced the pictures with the results from another replicate. We quantified the band density in the new figure according to another reviewer’s suggestion. In the new figure, the decrease of LSD1 and TRIM25 interaction were quite close. It indicated that the endogenous LSD1 has a more critical effect on TRIM25-RIG-I interaction than others.

10. The authors might show the localization of LSD1 and RIG-I in cells upon SeV infection to demonstrate these proteins can be colocalized in the physiological condition.

Response: As we have shown in Response Fig. 6, LSD1 is localized in both nuclei and cytoplasm in some cells.

Part III – Minor Issues: Editorial and Data Presentation Modifications

Please use this section for editorial suggestions as well as relatively minor modifications of existing data that would enhance clarity.

Reviewer #1: 1. It is quite clear from the data that LSD1, by silencing or overexpression, affected IFN/ISG expression (Fig 1). However, whether this effect was through specific regulation of RIG-I was insufficiently proven. Silencing LSD1 upon RIG-I or MDA5 overexpression should be performed to determine specificity. Relating to this point, in Fig 3C, MDA5 or MAVS should be included as controls to confirm RIG-I specificity.

Response: EMCV is known to be sensed by MDA5 but not RIG-I (2). We then used EMCV to treat cells and found that LSD1 knockdown did not affect IFNB1 expression induced by EMCV. When MDA5 and LSD1 were co-expressed in cells, we could detect the interaction between the two proteins, but LSD1 expression did not enhance K63-linked poly-ubiquitination on MDA5 (Response Fig. 10). Together with our data presented in the manuscript, these indicated that LSD1 does not regulate MDA5 polyubiquitination and the downstream pathway, and its effect is specific to RIG-I.

Response Fig. 10 (A) A549 cells were transfected with negative control siRNA (siNC) or LSD1 siRNAs (1#). The cells were infected with EMCV for 12h. The relative mRNA levels of *IFNB1, ISG54*, and *ISG56* were detected by RT-qPCR. (B) HEK293T were transfected with Flag-LSD1 and HA-MDA5 for 24h, followed by co-immunoprecipitation and immunoblotting analysis as indicated. (C) HEK293T were transfected with Flag-LSD1 and HA-MDA5 for 24h, followed by co-immunoprecipitation and immunoblotting analysis as indicated. (D) HEK293T were transfected with Myc-K63O-Ub and HA-MDA5 together with a control or Flag-LSD1 expression plasmid for 24h, followed by immunoprecipitation and immunoblotting analysis as indicated.

2. From the mapping data in Fig 3F, the conclusion in lines 225-227 stating LSD1 interacted weakly with ΔCARD seems incorrect. Full-length RIG-I, despite being expressed more than ΔCARD, bound even lesser to LSD1. How to explain this?

Response: We did not describe it accurately and have changed the description in the revised manuscript.

3. The ex vivo and in vivo data in Fig 6 convincingly show the antiviral effect of LSD1. It would be nice if the authors could perform additional data to link these data to RIG-I/TRIM25, for example by performing 1 experiment in cells from these mice. Moreover, why was the tail-intravenous route chosen over intranasal for VSV challenge? Were there any data on animal survival?

Response: We tried very hard to express TRIM25 in the cells derived from our mouse models, but failed to get satisfying transfection efficiency. Since it is well known that it is really difficult to transfect into primary cells, we tried some experiments to further clarify our model in human cells. We expressed TRIM25 or RNF135 in wild type or LSD1 knockdown cells, and found that TRIM25 could not ubiquitinate RIG-I without LSD1, but RNF135 did not change obviously (Fig. 5H). We further knocked down LSD1 in TRIM25 knockout cells, and found that LSD1 deficiency decreased RIG-I polyubiquitination and IFNB1 expression in wild type cells, but not in TRIM25 knockout cells (Fig. 5I&J). These indicated that LSD1 and TRIM25 are functionally related. Now we included these data into the revised manuscript. I hope the reviewer could understand our difficulty of the experiment.

Both tail-intravenous and intranasal routes are reported to be used for VSV challenge (3, 4), and we did not have any specific purpose here. During revision, we got data for animal survival (Response Fig. 11) and have added it into the manuscript as Fig. 6H. The result indicated that Lsd1 deficiency significantly decreased mouse survival rate upon VSV challenge.

Response Fig. 11 *Lsd1*fl/+ *Lyz2*-*cre*- and Lsd1 fl/+ *Lyz2-cre* + mice were intraperitoneal injected with VSV at 10^8 PFU per mouse (n = 10 for each genotype group). The survival rates of mice were recorded.

Reviewer #2: Fig. 2B/5F: the LSD1 KD efficacy is relatively low and varies a lot, please quantify for 3 independent replicates.

Response: We have quantified the protein level by scanning the original film and analyzing with ImageJ software. To save space, we just label the number of the presented blots in the figures (Fig. 2B&5F).

Fig. 2B Please remove the panel or at least add a VSV protein, not just GFP.

Response: We have removed the panel for GFP here as suggested.

Fig. 2 It would have been more convincing if a second virus would have been included.

Response: In the manuscript, we have used VSV and SeV for experiments. During revision, we have used EMCV to treat the cells. Since EMCV is mainly sensed by MDA5, but not RIG-I, we did not observe a significant effect for LSD1 in EMCV-induced gene expression (Response Fig. 10). There indicated that the effect we observed for LSD1 in sensing RNA virus is specific for RIG-I.

Fig3 A and B: Please quantify the levels of p-IRF3/IRF3 and p-TBK1/TBK1 of 3 independent experiments

Response: We have quantified the protein level by scanning the original film and analyzing with ImageJ software. To save space, we just label the number of the presented blots in the figures (Fig. 3A&B).

Fig. 3C: How can the authors say specifically, if only RIG-I was tested, was a larger panel checked?

Response: We did examine the interaction between LSD1 with other proteins in the pathway (Response Fig. 12). The results indicated that LSD1 interacts with RIG-I, but not VISA, TBK1 and IRF3.

Response Fig. 12 HEK293T cells were treated with SeV as the indicated time. Then the lysate was immunoprecipitated with anti-LSD1 and blotted with the indicated antibodies.

Fig 3F: Indicate that HA-LSD1 was overexpressed. Further, the LSD1 Input is not equal.

How can you explain that LSD1 interacts with Flag-CARD, but not the full-length RIG-I? Please at least address and discuss.

Response: Here, we still detected the interaction between full-length RIG-I and LSD1. Because we ran RIG-I full length and truncated proteins in the same blot and the signal for Flag-CARD was too strong, the band for full-length RIG-I seemed quite weak. We have corrected the text to better describe the result.

Fig 4E/F: Please quantify the ubiquitination levels of 3 independent experiments.

Response: We have quantified the protein level by scanning the original film and analyzing with ImageJ software. To save space, we just label the number of the presented blots in the figures (Fig. 4E&F).

Fig 4B: Please use another replicate where the actin level of the (-) K63 lane are not drastically lower

Response: We have replaced the Fig. 4B with a new figure, which was shown below (Response Fig. 13).

Response Fig. 13 Flag-RIG-I and HA-LSD1 were expressed in HEK293T with Myc-K48O-Ub or Myc-K63O-Ub. Then ubiquitination assay was performed to check RIG-I polyubiquitination.

Fig 5F: It seems like HA-RNF135 decreases LSD1 expression (there is nearly no LSD1 detectable in the Input). Was this effect observed in all 3 experiments or was it just observed on this occasion?

Fig. 5F: Bubble in blot at the crucial band, has to be replaced in a final version.

Response: The above blot was due to some experimental fault. We have replaced Fig. 5F with a new figure (Response Fig. 14).

Response Fig. 14 LSD1 was knocked down in HEK293T, and then Flag-RIG-I was expressed with its E3 ligases. Immunoprecipitation and western blotting were performed as indicated.

Fig. 6: Show survival of mice when challenged.

Response: During revision, we got data for animal survival (Response Fig. 11) and have added it into the manuscript as Fig. 6H. Lsd1 deficiency significantly decreased mouse survival rate upon VSV challenge.

Abstract line 25: RIG-I stands for retinoic acid-inducible gene I and not for retinoic acid-inducible I gene receptor

Response: We have corrected the text and figures as suggested.

Line 332: Lsd1 - LSD1

Line 340: Tbk1, Irf3 - TBK1, IRF3

Line 651: rig.i, Lsd1 - RIG-I, LSD1

Response: These stand for genes or proteins of in mouse cells. We think they should keep as what they are.

Please improve the consistency of the manuscript. Sometimes the IPs are labeled as IP: Flag (e.g. Fig 4D) and sometimes as Flag IP (e.g Fig 4F),

Response: We have corrected the text and figures as suggested.

qPCRs are labelled with either IFNB1.. (e.g Fig. 1) or Ifnb1…- (e.g Fig 6, Fig S3), WBs with either p-IRF3 (e.g. Fig 3) or p-Irf3 (e.g. Fig S3).

Please remove the overstatements in line 196, line 176

Response: These stand for genes or proteins of in mouse cells. We think they should keep as what they are.

Reviewer #3: 1. The materials and methods of the manuscript were not described clearly:

(1) The Sendai virus and GFP-tagged VSV were used in the research, but there is no description in the materials and methods. How much SeV was used for stimulation? How was the GFP-VSV generated or obtained?

(2) The source or reference for the siRNA library was not mentioned in the text.

(3) The reference for calcium phosphate transfection is suggested to be added.

(4) What kind of poly(I:C) was used in the research?

Response: We have added the requested information in the manuscript. Please refer to the Method section of the revised manuscript.

2. In the Fig. 5D, two bands were detected for TRIM25, as marked by the authors. Could the authors explain it?

Response: We have checked out previous results and literatures. It seemed that when over-expressed in cells, we can often observe two bands. It is possible that they are both TRIM25 protein and the upper band is possibly modified. We have removed the “*” in the blot. Interestingly, LSD1 or RIG-I seemed to only interact with one of them (Fig. 5E&F), suggesting another layer of regulation probably exists.

3. The authors should denote what the Myc-Ub-K63O is in the legends of Fig. 4E and Fig. 5G.

Response: We have added the description as suggested.

**Reference**

1.	Sheng W, LaFleur MW, Nguyen TH, Chen S, Chakravarthy A, Conway JR, et al. LSD1 Ablation Stimulates Anti-tumor Immunity and Enables Checkpoint Blockade. Cell. 2018;174(3):549-63 e19.

2.	Kato H, Takeuchi O, Sato S, Yoneyama M, Yamamoto M, Matsui K, et al. Differential roles of MDA5 and RIG-I helicases in the recognition of RNA viruses. Nature. 2006;441(7089):101-5.

3.	Brown CW, Stephenson KB, Hanson S, Kucharczyk M, Duncan R, Bell JC, et al. The p14 FAST protein of reptilian reovirus increases vesicular stomatitis virus neuropathogenesis. J Virol. 2009;83(2):552-61.

4.	Tomczyk T, Wrobel G, Chaber R, Siemieniec I, Piasecki E, Krzystek-Korpacka M, et al. Immune Consequences of in vitro Infection of Human Peripheral Blood Leukocytes with Vesicular Stomatitis Virus. Journal of innate immunity. 2018;10(2):131-44.
