# Supplementary material for: Histone demethylase LSD1 promotes RIG-I poly-ubiquitination and anti-viral gene expression
Source: PLoS Pathog. 2021 Sep 16;17(9):e1009918. doi: 10.1371/journal.ppat.1009918 (PMC8445485; doi:10.1371/journal.ppat.1009918)
Supplement: Attachment — Submitted filename: Rev2 letter to Editors.docx [file ppat.1009918.s013.docx]

Dear Editors,

We are submitting a revised manuscript entitled “*Histone demethylase LSD1 promotes RIG-I poly-ubiquitination and anti-viral gene expression*” (PPATHOGENS-D-21-00884R1) to *PLOS Pathogens.* We were encouraged very much by your positive response. Since we just need to answer several questions, we did not prepare a separate response letter and will address all the comments below.

(1) Much of the data provided in the response to reviewers is quite compelling, and I would suggest that it should be shown in the manuscript or supplemental data as it increases the rigor, significance, and impact. Specifically, I would suggest including Response Figure 1, 2B, 3, 4, 5, 7, 8, 10.

Response: We have added the figures into the manuscript as suggested. Specifically, we added Res. Fig. 1 as Sup. Fig. S1A-C; Res. Fig. 2B as Sup. Fig. 7B; Res. Fig. 3 as Sup. Fig. S7C; Res. Fig. 4 as Sup. Fig. S3C&D; Res. Fig. 5 as Sup. Fig. S7D; Res. Fig. 7 as Sup. Fig. S8A-C; Res. Fig. 8 as Sup. Fig. S4A-C; Res. Fig. 10 as Sup. Fig. S2A-D.

(2) In addition, please add a discussion of how the cytoplasmic RIG-I and the often nuclear LSD1 could be interacting to the discussion. I don’t think that you need to show the data of Response Figure 6, just describe what might be happening or that you acknowledge that its perplexing and/or interesting.

Response: We have discussed the above result in the discussion section, at page 16 of our revised manuscript.

(3) A discussion of how binding and ubiquitination of RIG-I by LSD1 are two separate events should be included, either in the results (lines 290) or as a point in the discussion, as this important point could be confusing to some.

Response: We have added it in the results, line 303 of the revised manuscript.

(4) Thank you for including the quantifications in Fig. 2B, 3A, 3B, 4E, 4F, 5F. Please show the SD of the quantifications of all three replicates to give the reader an idea of the variation among replicates (for example, 0.63-/+ .2).

Response: Since it is not much space in the figures, we showed the results as histograms. The corresponding data are shown in Fig. 2C, Sup. Fig. S1D&E, S3A&B, & S7A.

Besides the figures, we have also added new text into the manuscript to describe the new results. Two manuscript files were prepared, one with all the corrections highlighted and one not. We hope the above corrections have meet your requirements and are looking forward to your response. Please feel free to let me know if more changes should be made.

Best regards,

Sincerely yours,

Min

Min Wu, Ph. D

Professor, and Director of

Department of Biochemistry and Molecular Biology

College of Life Sciences, Wuhan University,

Wuhan, Hubei, China, 430072

Email: wumin@whu.edu.cn
